# Brexit’s Implications for the Transatlantic Relationship

**DOI:** 10.1134/S1019331622080093

**Published:** 2022-06-29

**Authors:** O. V. Prikhodko

**Affiliations:** Institute for the U.S. and Canadian Studies, Russian Academy of Sciences (ISKRAN), 121069 Moscow, Russia

**Keywords:** Brexit, United States, Britain, European Union, transatlantic relationship

## Abstract

Since the Second World War, the United Kingdom has served as a transatlantic bridge between the United States and Europe and as a conduit of the US influence in European affairs. Since joining the EU, Britain has been one of the main contributors to the European Union’s foreign, security, and defense policies. The withdrawal of the United Kingdom from the EU in 2020 marked a milestone in European politics. The UK’s decision to leave the European Union has raised questions concerning probable implications of Brexit for the transatlantic relationship and the European balance of power. Brexit entails multifaceted changes in Britain’s global posture, in particular, new nuances in its dealings with the United States and European partners. These shifts embrace a wide range of political, defense, security, and economic issues. They have stirred up debates on the British strategy’s tilt towards the Indo–Pacific and Britain’s future cooperation with its American ally and the EU. Having considered developments in the Washington–London–Brussels relations in the wake of the Brexit referendum, this article figures out trends in the interactions of these key players that reflect their visions of a post-Brexit reality.

## INTRODUCTION

In June 2016, the UK held a referendum in which the majority (51.9%) voted for the country’s exit from the European Union. On January 31, 2020, the United Kingdom officially left the EU. In December of the same year, the parties signed an Agreement on Trade and Cooperation and several additional documents (on the use of nuclear energy for peaceful purposes, the exchange and protection of classified information, etc.), which govern their relationship in various fields except for foreign policy, military security, and defense. By January 1, 2021, the transition period ended, during which European legislation on the EU single market and customs union continued to operate on British territory. Brexit, from the point of view of its supporters, is intended to prove that the United Kingdom, freed from the EU shackles, can succeed much more in “free floating” than remaining in the European Union, which they call a “totalitarian superstate.” The UK is the fifth largest economy in the world in terms of GDP [1] and the leading European military power. Britain’s exit from the EU will weaken integrated Europe, which many politicians and experts in the West perceive as a blow to the liberal world order, because the European Union serves as its stronghold in the European space from Lisbon to Riga.

*Brexit provoked a sharply negative reaction from the US and European liberal elites because it is a victory for the principle of the supremacy of state sovereignty, which is contrary to the idea of globalization, which disregards national borders.* The growth of electoral support in Europe for nationally oriented, antiglobalization sociopolitical forces was caused by the global crisis of 2008, the collapse of the “open door” immigration policy, and the aggravated socioeconomic problems generated by globalization. Brexit reflects this general trend. According to a number of Western political scientists, when analyzing the causes of the erosion of American hegemony, “the role of transnational anti-order movements in Europe and North America received comparatively little attention” before Brexit and Donald Trump’s win in the 2016 elections (Cooley and Nexon, 2020, p. 14).

The Brexit saga unfolded under three American presidents: Barack Obama, Donald Trump, and Joe Biden. The political and ideological views of these leaders and the circles of the US ruling elite behind them largely determined the nature of Washington’s response to the UK’s separation from the European Union. However, in the assessments of Brexit by Democrats and Republicans, common typical features are visible, reflecting the deep interests of the American state, which remain intact with a change of administrations.

## US RESPONSE TO BREXIT

The American perception of Brexit has been shaped by the interplay of numerous factors. The unique ties of alliance between the United States and Britain occupy a special place among them. The American–British axis is one of the main pillars of the Western world order. The signing by the leaders of these two countries of the New Atlantic Charter in June 2021 was a clear confirmation of this. Washington views the United Kingdom as its most important ally and conductor of its influence in international affairs, especially in Europe, because the views and approaches of the two countries are close to each other’s or coincide. By cultivating a special relationship with Britain, US administrations encouraged Britain to view the United States as a more important partner than the European powers or the EU. These ties have “allowed the United States to keep a foot in Europe, which has become increasingly relevant in the course of the United States[’] pivot towards [the] Asia–Pacific and also enabled the United Kingdom to play a more profound role within the Atlantic Alliance” (Ewers-Peters, 2021, p. 579).

During periods of tension in transatlantic relations, the ability of the United Kingdom to function as a link between North America and Europe, and between NATO and the EU, was of great value to the United States. A trusting relationship with London became a particularly valuable asset in American politics when it acted as a leader in the EU’s diplomatic and security policy. Washington capitalized on British EU membership, using it to shape the foreign policy priorities and military plans of a united Europe. It supported the desire of London to tune European defense cooperation to the goals of NATO (United States). As the English political scientist W. Rees noted, “lacking the ability to steer European integration, the United States relied upon the United Kingdom to constrain the EU in ways congruent with its interests.” He stated that “America’s promotion of bilateral security and defence policy cooperation with the United Kingdom was conducted at the expense of both countries’ multilateral relationships with the EU.” America pursued a strategy that aggravated friction between its two key Atlanticist institutions, NATO and the EU (Rees, 2017, pp. 561, 565). The UK has strongly opposed any initiatives aimed at forming the EU as an independent center of power and geopolitical influence. It has resisted French attempts to compete with the United States for leadership in Europe or to question NATO’s preeminence in the European security system.

The special relationship with the United States and EU membership opened up opportunities for London to mediate between Washington and European partners in transatlantic disputes. According to the American historian A. Cyr, Britain’s role as a mediator between the United States and Europe has acquired particular value in the “context of friction involving President Donald Trump, German Chancellor Angela Merkel, and others in Europe” (Cyr, 2018, p. 92). *However, Brexit effectively deprives the UK of its ability to mediate and limits its contribution to transatlantic security cooperation to NATO.*

The benefits to American interests from UK remain in the European Union were not limited to the security sphere. Former US Ambassador to the EU (2014–2016) A. Gardner, in his memoirs, notes the important role of the British government in reaching, in the summer of 2016, a US–EU agreement on regulation in the field of the digital economy and the protection of personal data, including the Data Privacy Shield agreement and proposals for the formation of a single market for EU digital services, which were relatively favorable for US companies (Gardner, 2020, p. 90).

The topic of Brexit emerged in the transatlantic agenda at the final stage of Obama’s presidency. The US administration supported the opponents of UK’s exit from the EU. On the eve of the British referendum, which took place on June 23, 2016, President Obama, Secretary of State John Kerry, and other American officials, in an attempt to influence the outcome of the vote, warned about the risks and possible negative consequences of Brexit. Particularly unpleasant for the US administration was the fact that *the Brexiteers in their campaign actively promoted the theme of the restoration of the national sovereignty of Britain, which ran counter to the concept of globalization promoted by the US liberal elite.* Gardner recalls that, like many in the Obama administration, he simply could not understand “the repeated argument that Britain had become a vassal state and that it was necessary to ‘take back control’ over borders, money, and laws.” In his opinion, this statement misled not only many voters in the United Kingdom but also key figures in the Trump administration. The US President “has referred to the EU as an ‘anchor’ around the UK’s ankle” (Gardner, 2020, pp. 74, 75).

The Obama administration actively promoted its position to the British side, including during contacts with prominent political figures from the Brexit camp, such as London Mayor (and future Prime Minister) Boris Johnson, who, speaking in the spring of 2016 in a popular TV show on British television, called the EU “a jail with the door left open” [2]. Washington did not skimp on words, condemning the “seditious” idea of the UK leaving the EU. However, its rhetoric could not erase from the memory of the British the harsh American criticism of various aspects of the activities of the European Union. Some Western analysts believe that the US itself unwittingly had a hand in Brexit, ostracizing the political ambitions of the EU and mercilessly criticizing Brussels for the failures of European policy on immigration and countering terrorism. This criticism played into the hands of the Brexiteers, and “among those advocating withdrawal were senior figures in the British security services who questioned the value of the EU in fighting terrorism” (Rees, 2017, pp. 563–564).

*The American political and expert community split into two camps in their assessment of Brexit.* The liberal wing expressed support for opponents of the UK’s exit from the EU, pointing to the potential losses and risks associated with this decision, in particular, the reduction of British influence in matters of international security and finance. Opposite views were held by right-wing conservative circles. They welcomed Brexit based on their conviction that a weakening of the European Union was in the American interest. For them, the European Union was a rival to the United States in many respects.

The results of the referendum, in which most Britons (51.9%) chose to leave the EU, caused undisguised disappointment in Washington. The liberal elite and think tanks affiliated with the Democratic Party perceived the outcome of the vote as a manifestation of an alarming trend in the rise of populist sentiment and as an indicator of the frustration of a large part of British society with the results of globalization and unwillingness to come to terms with the country’s postimperial status. According to the American political scientist G. Wilson, in circles close to the Obama administration, Brexit “reflected both the disturbing rise of authoritarian populism and the inability of the British to adapt to modernity” (Wilson, 2017, p. 553).

Although Washington and Brussels characterized the bilateral relationship as a partnership, on some issues the EU created problems for American policy, and the US often used its ties with the UK to solve them. As Gardner notes in his book, given that the United States and the United Kingdom “see eye to eye on nearly every foreign, economic, and security issue, it is natural that Washington would want the UK ‘inside the EU tent’ influencing EU decision-making and making the EU more economically liberal, Atlanticist, and pro-NATO” (Gardner, 2020, p. 88).

From the point of view of the American Democrats, Britain leaving the EU was a sensitive blow to the liberal world order, having a negative impact not only on both parties involved but also on Europe as a whole, provoking political processes that cause concern in it. The British referendum gave impetus to populist movements in Europe, especially in France, the Netherlands, and Denmark, which could lead to a split and potentially disintegration of the EU. Brexit exacerbated the internal problems of the EU; as a result, their European partners, as Washington feared, could immerse themselves in their solution for a long time, devoting fewer forces and resources to interact with the United States in international affairs. Commander-in-Chief of US Forces in Europe, Lieutenant General F. Hodges, who retired in December 2017, issued a warning at the time, saying that the disintegration of the EU could have serious consequences for NATO as well.

Speaking in the spring of 2016 at a hearing in the Select Committee on Brexit of the House of Commons of the British Parliament (where he was invited to present the position of the Obama administration), Gardner explained why Washington was in favor of maintaining Britain’s membership in the EU. The core of his explanation was as follows: “Having the United Kingdom in the European Union gives us much greater confidence about the strength of the transatlantic union” (Gardner, 2020, p. 84). From the point of view of the American establishment, Brexit weakens the position in Europe of a group of countries that traditionally tend to having closer ties with the US and Britain in their policies. Washington has made no secret of its concerns about the possible negative impact of Brexit on security, given that the UK has traditionally played an important role in defense cooperation within the European Union.

One of the reasons for the American concern is the risk of the unbalancing of integrated Europe after the British withdrawal. Brexit upsets the existing balance in the EU, which relied on the big European troika: Germany, France, and Britain. Brexit will result in increased German influence, while Britain is traditionally considered by American strategists as a counterbalance to German power.

The United States does not exclude *tangible economic consequences for transatlantic relations*, believing that Brexit could move the EU to a more protectionist position in trade negotiations, strengthen the influence of those circles in Europe that protect the interests of leading national companies, and promote the ideas of European industrial policy and planning, narrowing the boundaries of free trade competition and the open market. The negative attitude of the Obama administration to Brexit was fueled by fears that the UK leaving the EU would harm the interests of American companies operating in both the manufacturing and services sectors (especially financial ones), which chose to be in the UK largely because it serves them as a springboard for entering the much larger common market of the European Union.

The victory in the 2016 presidential election of Trump, who made no secret of his support for Brexiteers, helped avoid a delicate situation in US–British relations, given that the Democratic candidate Hilary Clinton criticized the results of the British referendum. Trump regarded Brexit as a rejection by the British of the “false idea” of globalization, and the weakening of the EU and the problems that have arisen in relations between London and Brussels as a win for the United States. He also considered acceptable a hard version of Brexit—the UK’s exit from the EU without an agreement. Trump was scathing about the efforts of then Prime Minister Teresa May, who tried to reach an agreement with the EU on terms that would have allowed as many British–European ties as possible to be preserved after Brexit. Western analysts noted that “in contrast to previous US administrations, however, the Trump administration did not think of the EU as a constraint on Berlin. Instead, it saw the EU as a mechanism to further German interests and power and even supported anti-EU initiatives and movements, including Brexit” (Simón et al., 2021, pp. 98–99).

A positive assessment of Brexit is widely spread in conservative American think tanks. According to experts from the Heritage Foundation and the American Enterprise Institute, Brexit will allow the UK to regain its independence and create opportunities for a new upsurge in US–British relations. The views of right-wing conservative circles are largely accounted for by their perception of the EU as a tool that Germany and France use in “unfair” competition with the United States.

The current position of official Washington is determined by the fact that President Biden and influential figures in his entourage, who worked in the Obama administration, stick to their negative attitude towards Brexit. Brexit is ushering in changes that may reduce Britain’s contribution to solving European problems affecting US interests. The Biden administration is interested in ensuring that the separation of the UK and the EU does not entail painful consequences. In the first year after Brexit, disagreements between London and Brussels came to the fore over specific but politically sensitive issues, such as the Northern Irish Protocol and fishing rights in British coastal waters. Although these disputes do not pose a threat to the unity of the West, which would require decisive steps from Washington, according to some Western political scientists, the Biden administration is not making enough efforts to help overcome the emerged disagreements and contribute to a mutual understanding between the UK and Europe.

## FOREIGN POLICY CONSEQUENCES OF BREXIT

The withdrawal of the UK from the European Union is a major international event that concerns various aspects of transatlantic relations. *In relation to America, Brexit is resulting in an even stronger strategic rapprochement between Britain and the United States.* The tilt towards closer cooperation with the United States can be seen in the updated strategy that Prime Minister Johnson presented to Parliament in March 2021. Participation in the creation of the triple alliance AUKUS, which was prepared secretly from the EU, the designation of the Indo–Pacific Region as one of the main priorities in the field of security and a number of other political innovations indicate that the post-Brexit Britain in its international positioning is actively adjusting to the strategic objectives formulated by the Biden administration.

The updated British strategy makes cooperation with the United States and NATO the highest priority, indicating the continuity of the basic foreign policy postulates of the United Kingdom, which do not alter with the change of governments. The document confirms the commitment to maintaining a special relationship with the United States, which is characterized as their “most important strategic ally” [3]. In matters of collective security, London intends to continue to rely on NATO, considering the Euro–Atlantic macroregion as the main area for its efforts. The assessments of global threats and trends contained in the strategy largely coincide with the analysis of shifts in international relations, which is given in American official documents—the annual report of the Director of National Intelligence (*Annual Threat Assessment of the U.S. Intelligence Community 2021*) and the four-year review of the American Intelligence Community (*Global Trends 2040: A More Contested World*).

Brexit promises certain benefits for American policy. *First,* in situations that cause disagreements between the United States and integrated Europe, the potential solidarity of Britain with European powers (as was the case with the Iranian nuclear deal in Trump’s presidency) will be unlikely achievable due to the lack of a legal and institutional basis for foreign policy cooperation between the United Kingdom and the European Union. London, freed from the obligations associated with EU membership, can now openly unite with the United States in opposition to European projects, which, from the US–British point of view, pose risks to the cohesion of the Atlantic Alliance. Britain supports American objections to the concept of European defense, which has been discussed within the EU for several years, considering the very idea of the strategic autonomy of Europe, which is the basis of the concept, to be harmful.

The vacuum of legal and institutional mechanisms for regulating British–European relations in the foreign policy sphere, emerged after Brexit, only strengthens the emphasis in London’s policy on closer cooperation with the United States. According to Western analysts, Anglo–American cooperation in intelligence and other areas of defense policy can be expected to expand and intensify after Britain’s exit from the EU (Cyr, 2018, p. 93). Admittedly, not everyone in the ruling Conservative Party agrees with such a bias in London’s strategy: there are those in the ranks of the Tories who believe that American and British interests may not always coincide, and London should collaborate more closely with its European partners on European security issues.

*Second,* one of the consequences of Brexit was the intention of the Johnson government to expand British involvement in the affairs of the Indo–Pacific Region (IPR), which corresponds to the current priorities of the American strategy aimed at containing China as the main systemic adversary of the West. The EU strategy for the IPR, published in September 2021 [4], although it notes some deterioration in relations between the EU and China, uses more diplomatic and balanced definitions in comparison with the assessments given to Beijing’s policy by officials in Washington and London. The EU is focusing on finding opportunities for cooperation with China, not on containing it.

*Third, Brexit raises the profile of NATO as a tool of interaction between the UK and their European partners in the field of defense and security, and any strengthening of the Atlantic Alliance is in the interests of the United States.* The EU curtailed the exchange of classified data with London, which was carried out through a special mechanism of the European Union. Britain lost access to some services of Galileo, the European satellite navigation system. The conditions for the participation of British contractors in projects implemented through the European Defense Agency (EDA), as well as in the framework of in-depth military-political cooperation under the auspices of the EU PESCO (Permanent Structured Cooperation) have become more complicated. In the preexit era, the UK acted as a link between NATO and the EU in some matters of transatlantic cooperation. Now these opportunities has considerably narrowed. European political scientists who tend to exaggerate the importance of the EU see Brexit as a weakening of NATO (Biscop, 2020, p. 90).

Britain’s withdrawal from the European Union did not cause significant changes and legal consequences in the relationship between the United States and the EU. The absence of mutual legal obligations in the sphere of bilateral political relations between the United States and the European Union facilitates the mutual adaptation of the parties to the consequences of Brexit. Interaction between Washington and Brussels on foreign policy issues is carried out through informal mechanisms and in formats that do not require the creation of a contractual legal basis for them, providing the parties with freedom in decision-making.

The Biden administration is interested in British–European cooperation in the field of defense and security since the fruitful interaction between Brussels and London would have a positive impact on both NATO–EU relations and the transatlantic community as a whole. Some Western experts, in particular former British Ambassador to the United States N. Sheinwald, believe that an important criterion for the utility of the UK for American policy in the eyes of the Biden administration will be the extent to which London will be able to establish cooperation with the EU after Brexit [5]. However, the Johnson government, not expecting tangible benefits from interaction with the European Union in the field of external security and international policy, rejected the proposal of Brussels to conclude an agreement. The previous cabinet, led by May, sought to achieve a legally binding agreement with the EU in this area, believing that it would be better to keep abreast of EU initiatives, ensuring their compatibility with NATO tasks, than to distance themselves from Brussels, giving it full carte blanche. The British rejection of the agreement with the EU undermined Washington’s hopes that the United Kingdom, after leaving the EU, would retain access to the mechanism for preparing foreign policy decisions in Brussels, which would allow incorporating the US–British point of view into this process.

*Britain is not showing any interest in establishing military-industrial cooperation with the EU.* It sees no tangible benefits for itself from the military-technical cooperation that the European Union, whose competence in such matters is very limited, can offer it. However, this does not exclude the development of military-industrial cooperation between Britain and leading European states on a bilateral or multilateral basis outside the European Union, especially when such interaction is consistent with NATO’s plans. Judging by the strategy for the modernization of the armed forces, which the British Secretary of State for Defense B. Wallace presented to Parliament in March 2021, the UK considers France as its most important partner in the EU [6]. London expressed its readiness to closely cooperate with Paris on military issues, including in regions of mutual interest—the Western Balkans, Iraq, and the Sahel.

Britain is one of the five leading military powers in the world. According to the American political scientist M. Beckley, the European Union’s deepened defense cooperation (PESCO) “is unlikely to offset the damage done by Britain’s exit from the EU, given that Britain accounted for a quarter of EU defense spending and half of EU military R&D spending” (Beckley, 2018, p. 107). On the one hand, Brexit creates uncertainty around the future of European military-technical projects that were initiated with British participation. On the other hand, the political obstacles that arose due to the inflexible position of London against the expansion of the EU’s competence in matters of defense cooperation are disappearing. Britain resisted the implementation of ideas aimed at deepening defense integration within the EU, blocking initiatives that could call into question American leadership in NATO. In 2003 and 2011, London vetoed proposals to form a centralized command and staff structure within the EU, seeing them as risks for the Atlantic Alliance. The United States has always supported its British ally in its vigorous opposition to the concept of a “European defense alliance.” According to Rees, “Brexit offers Paris the opportunity to realise objectives that seek EU autonomy from American power” (Rees, 2017, p. 568).

Having lost access to the decision-making mechanism in the EU and having lost the function of the “transatlantic bridge,” Britain keeps negligible opportunities to project the US and NATO interests in the European Union. However, the EU is also losing a significant part of its potential with the departure of one of the key players that made a great contribution to European structures dealing with defense and security issues. According to Western political scientists, in particular N. Ewers-Peters, Brexit may negatively affect the effectiveness of cooperation between NATO and the EU and slow down the interaction of these organizations (Ewers-Peters, 2021, p. 588). The foreign policy resources of the European Union are also being reduced, given that the extensive network of world-wide links of British diplomacy will become inaccessible to Brussels.

Brexit changes the usual alignment of forces in integrated Europe. The challenge for the United States is to determine how the new EU geopolitical configuration may affect American interests. Washington is compelled to realize the decline of British influence in European affairs and to take into account the opinion of Germany and France to a greater extent when considering European problems. During the Merkel era, Germany played a key role in determining the strategic prospects for an integrated Europe, achieving a balance between the competing interests of countries, and maintaining the unity of the EU on key issues. Brexit only reinforces Berlin’s status as the architect of European compromise. The states of Central, Eastern, and Northern Europe often, when defending their position, especially in matters of security and migration, turned to the UK for support, with which they have shared views. Now these countries have no counterbalance to German power in the European Union.

The decline of British influence in European affairs is forcing the United States to build relations with Germany more carefully and to be more attentive to its interests, given the increased political weight of Berlin in the European balance of power after Brexit. Formulating its approach to doing business with the EU, Washington has always been aware that the German position is often the determining factor in shaping the policy of an integrated Europe. The decisions of the governing bodies of the EU, as a rule, reflect the priorities of the FRG. Many areas in the activities of the European Union require financial support, and Germany is the major donor that provides the largest contribution into its budget.

According to Western political scientists, despite the fact that American attention is increasingly focused on China and the IPR, Washington “will likely seek engagement in Europe that is sufficient to influence the strategic interaction between Germany and Russia” (Simón et al., 2021, p. 100), even if these efforts require quite a lot of political or other costs from it. The concessions of the Biden administration on sanctions against Nord Stream 2 just demonstrated the importance for it of maintaining good relations with Berlin. Washington considered it possible to show flexibility on this issue, given the fact that Brexit did not lead to a weakening of the EU sanctions’ pressure on Russia. The UK has often acted as a mastermind of the European sanctions policy directed against the Russian Federation, and, being outside the EU, it is not reducing its activity in this matter.

## IMPACT OF BREXIT ON TRANSATLANTIC RELATIONS IN THE TRADE AND ECONOMIC SPHERE

The UK officially left the EU on January 31, 2020, and after another 11 months, the transition period ended, and European legislation on the common market and the customs union finally lost force on its territory. At the end of December 2020, the United Kingdom and the EU entered into a Trade and Cooperation Agreement (TCA), which provides for duty-free and quota-free access to each other’s markets. Critics of Brexit regard it as a weak and rather painful deal for the British economy. Suffice it to say that the financial services sector, which is of great importance to the economic prosperity of the United Kingdom, retains access to the European single market for only a very limited list of activities.

*Brexit has caused multidirectional economic consequences that affect not only Britain itself but also the United States and the European Union.* Since 2018, when the decoupling process became irreversible, there has been a steady decline in the volume of UK trade with the EU. In 2020, British merchandise exports to the EU countries decreased by $40.5 billion (17.8%) compared to two years previously to $187.2 billion. Imports diminished during this time by $58.7 billion (16.5%) to $297.6 billion [7], but in absolute terms it still represents an impressive value.

The opposite trend was observed in UK trade with the United States. British exports to the United States have grown steadily, from $59.1 bln in 2017 to a record $73.5 bln in 2019. However, they fell to $55.6 bln in 2020 as a result of lower business activity on both sides of the Atlantic due to the coronavirus pandemic. In these years the volume of British imports from the United States grew even faster. As a result, the trade surplus in favor of the United States in bilateral trade reached a historically high level of $8.8 billion [8]. In the first ten months of 2021, US exports of goods to the UK amounted to $50.9 billion, and imports, to $46.1 billion [9], and, judging by the current dynamics, the volume of bilateral trade is unlikely to exceed the prepandemic record this year.

Brexit accelerated China’s rise to first place in the list of leading trading partners of the United States, pushing the EU to second position, as the EU statistics no longer take into account the UK’s trade volume with the United States. According to our calculations, in 2020 the share of the EU in British exports was 52.7%, while the share of the United States was 14.1%. *This shows the unequal importance for the UK of the markets of its two main trading partners. Commercial ties with the EU continue to play a paramount role in the British economy, despite Britain’s withdrawal from the European Union.*

Access to the British market is of no small importance, especially for those US companies and corporations that operate in the automotive industry, financial services, and insurance; these are the whales of American business such as Ford, General Motors, City, J.P. Morgan Chase, Goldman Sachs, Citigroup, Morgan Stanley, and Bank of America. The main investors in the British economy are large nonbank holding companies, financial and insurance firms, and manufacturing corporations. The accumulated American direct investment in the UK was $851.4 billion as of 2019 [10].

According to US statistics, the United Kingdom ranks seventh among the largest trading partners of the United States. American business has traditionally considered the UK a profitable place to allocate capital, except for a number of sectors of its economy where there were restrictions established by EU law. With its withdrawal from the European Union, the investment attractiveness of Britain for American business has declined: such an advantage as the possibility of entering the common European market on favorable terms through the “British gate”—joint ventures and British subsidiaries of American firms and corporations—has disappeared. As a result, Ford has reduced planned investment in its production facilities in the United Kingdom. Other American companies have also revised their investment plans.

The report of the US Trade Representative K. Tai notes that during the period of the UK’s membership of the EU, American exporters and investors faced obstacles when they entered the UK market and when they tried to maintain or expand their presence in certain sectors of the British economy. Many of these obstacles have persisted beyond January 1, 2021, as the UK continues to comply with EU regulations [10].

US–British trade and economic relations before Brexit were governed by the US–EU Trade Agreement. When the UK ceased to be part of the EU, Washington and London faced the need to regulate their trade relations on a new basis. London is unlikely to be able to achieve for itself such favorable conditions as those that will be spelled out in the US–EU Free Trade Agreement, because it has incomparably fewer opportunities than Brussels to influence Washington’s position.

The Biden administration confirmed that Britain is the closest ally of the United States, but at the same time made it clear that it would firmly defend American interests in the negotiations. Signals coming from Washington suggest that a comprehensive free trade agreement with the UK is not on its list of priorities. The United Kingdom accounts for 2.6% of US foreign trade and 3.6% of US exports [11]. As can be seen from these indicators, from a purely economic point of view, the importance of trade and economic ties with Britain for the American economy is not that great. However, in a number of cases, these ties are exclusive, which, combined with the special relationship between the two countries in the field of security, significantly increase their worth compared to the nominal value of the trade turnover.

Negotiations on a trade agreement between the United States and the United Kingdom began on May 5, 2020. The parties have made notable progress in agreeing on certain provisions of the trade agreement. In addition, they have entered into five separate regulatory standards agreements, identical to those in force between the US and the EU. These standards embrace product groups such as wine, distilled spirits, marine and telecommunications equipment, electromagnetic capability, pharmaceutical products, and they also cover insurance.

However, the stumbling block in US–British free trade talks is a disagreement over the conditions for allowing US farm products to enter the UK market. This applies primarily to GMO products and meat products made from raw materials in the production of which hormones are used. There is also no agreement among the parties regarding the standards applied to food products. To protect its market, the UK maintains high tariffs on certain agricultural products, especially fish and seafood, as well as trucks, passenger vehicles, and wood products. The issue of a tax on US companies that operate in the British digital services market remains unresolved.

To speed up the conclusion of a free trade agreement with the United States, the UK can soften the terms of access to its market, making it more attractive to the American companies. However, such compliance will make it difficult for London to conclude free trade agreements with third countries that will demand no less favorable terms for themselves. In addition, the separation of the British regulatory system from EU norms would have negative consequences for its economy, far exceeding the positive effect of the free trade regime with the United States.

The situation around the British Internal Market Bill showed that the Biden administration is ready to intervene when British–European disagreements may develop into a conflict. The document contained provisions on UK customs control, which contradicted the agreement between Brussels and London on the terms of Brexit. Washington warned the British authorities about the inadmissibility of establishing border and customs controls between the Republic of Ireland and Northern Ireland, as this would violate the 1998 Belfast Agreement reached with American mediation on the settlement of the Northern Ireland conflict. Under American pressure, the Johnson government agreed to amend the bill.

The change in the border and customs control rules caused by Brexit has led to a complication of the situation with the delivery of export goods from EU countries to Northern Ireland, which remains in the EU market space. A compromise on this issue is recorded in a special protocol, which is part of the agreement on the terms of the UK’s exit from the EU. However, the problem of customs control between Britain and Northern Ireland has not been fully resolved. Although there has been some softening of London’s position lately, it is still far from the complete elimination of differences. Washington calls on London to refrain from steps that could destabilize the situation in Northern Ireland. With differences between London and Brussels persisting, US mediation becomes almost inevitable, given that the US is the guarantor of the 1998 Belfast Agreement. British–European disputes over sensitive issues provide Washington with an opportunity to act as an arbiter, which increases American influence in European affairs.

## CONCLUSIONS

The United States views Britain as its most important ally, and any significant changes in the country’s geopolitical position, especially those that affect the balance of power in Europe, are subject to scrutiny in Washington. However, the significance of Brexit for transatlantic relations should not be overestimated: the UK’s exit from the EU does not disrupt the functioning of the Western world order. Brexit, which is the product of a unique combination of objective and subjective reasons, does not pose a strategic challenge for the United States, although it affects American interests in various areas. It will not be able to shake the position of the United States in Europe, even if the EU’s movement towards “strategic autonomy” accelerates after the UK’s withdrawal from the European Union.

Brexit has a multidirectional impact on the transatlantic relationship. It is leading to changes to American policy in relation to Europe and it has increased the value of cooperation with Germany and France for Washington. Brexit narrows the American possibilities of influencing the EU on internal processes in integrated Europe and on the international policy of Brussels. If London continues to shy away from political cooperation with the EU, and the strategy of the Johnson government provides a basis for such an assumption, its value as a partner of the United States in European affairs will be diminished. At the same time, having got rid of the restrictions imposed by EU membership, London has more freedom to act in support of Washington.

The Biden administration is interested in establishing constructive cooperation between London and Brussels, since the rise of competition among the allies, and even more so their mutual alienation, would hinder American efforts to consolidate the West. In a situation of estrangement between Britain and continental Europe, the United States would need additional forces to maintain the European balance, which would interfere with the concentration of American resources to counter the “Chinese challenge.” The distancing of the UK and the EU from each other is an unfavorable scenario for American policy, as it carries an increased risk of friction and conflict between the European partners of the US.

Brexit removes restrictions on defense projects within the European Union, which arose because of the British opposition supported by the United States: Washington and London are interested in slowing down the military-political integration of the European Union, fearing that it will become a competitor to NATO. At the same time, the withdrawal of the United Kingdom from the EU reduces the military potential of the “strategic autonomy” of Europe: the EU cannot, as before, count on British participation in its operations. The weakening of European military-technical and military-industrial cooperation as a result of the UK leaving the EU is in the interests of the United States in terms of the race for global technological leadership and in the context of competition between American companies and European manufacturers in the international arms market.

The United States is facing controversial consequences of Brexit. On the one hand, there is an *“Americanization” of British foreign policy* as Britain has been released from many of its obligations within the EU, which is expressed in its more active and large-scale participation in American efforts aimed at countering Russia and China in strategically important regions of the world. Britain is one of the key partners of the United States in efforts to strengthen the eastern flank of NATO. In September 2021, it cofounded the AUKUS trilateral alliance, demonstrating its willingness to help Washington in every possible way implement its defense and geopolitical projects in the IPR.

On the other hand, for the United States Brexit means the loss of a powerful channel for projecting American and NATO influence within an integrated Europe. It will become more difficult for Washington to work with Brussels to mobilize the political, economic, and technological resources of the EU for the fight against China and for other goals on which the views of the parties do not coincide. London is largely deprived of the opportunity to act as a mediator between NATO and the EU: in the past, British diplomacy, performing this function, had repeatedly helped mitigate the differences that arose between the two organizations, especially when the ambitions of the United States and France clashed.

Despite the disappointing outcome of the Brexit referendum, the Obama administration in 2016 and five years later, the Biden administration confirmed the preservation of the “special” relationship between the US and the UK, which, however, did not help London achieve favorable terms on its withdrawal from the EU. *Brexit highlights the exclusive nature of the US–British “special” relationship.* Cooperation with the United States is becoming even more important for the UK in terms of national security interests and its status in international politics. It follows from the updated British strategy that, having left the EU, the United Kingdom will actively cooperate with the United States regardless of the opinion of its European partners. This, of course, does not mean that the UK is deliberately moving away from integrated Europe, but it is unlikely to be able to maintain its former involvement in European affairs not related to security.

It is important for the Biden administration that Brexit does not cause destabilization in the transatlantic community. Washington sees the security and stability of a united Europe as a prerequisite for concentrating Western forces and resources on containing Russia and countering China. Washington believes that tensions in inter-European relations may make it difficult to achieve American foreign policy goals. Internally divided and mired in its problems, Europe (EU) is not capable of providing significant support to the United States in international affairs, whether it be in Europe or the Indo–Pacific Region.

## SOURCES

1. World Bank. Gross Domestic Product 2020 ranking. https://datacatalog.worldbank.org/dataset/gdp-ranking. Cited November 20, 2021.

2. A. Withnall, “EU referendum: Boris Johnson calls the EU ‘a jail with the door left open’ and says it would be ‘wonderful’ to leave,” Independent on-line, Mar. 6 (2016). https://www.independent.co.uk/news/uk/politics/boris-johnson-says-eu-jail-door-left-open-uk-leave-a6915241.html. Cited October 5, 2021.

3. Global Britain in a competitive age: The integrated review of security, defense, development, and foreign policy. https://assets.publishing.service.gov.uk/government/uploads/system/uploads/attachment_data/file/969402/The_ Integrated_Review_of_Security__Defence__Development_and_Foreign_Policy.pdf. Cited August 11, 2021.

4. The EU strategy for cooperation in the Indo–Pacific, Brussels, September 16, 2021. https://ec.europa.eu/info/sites/default/files/jointcommunication_indo_pacific_ en.pdf. Cited November 20, 2021.

5. CER podcast: Biden vs Trump: America’s choice matters to Europe, October 28, 2020. https://www.cer.eu/media/cer-podcast-biden-vs-trump-americas-choice-matters-europe. Cited August 11, 2021.

6. Defense in a competitive age. https://www.gov.uk/government/publications/defence-in-a-competitive-age. Cited October 1, 2021.

7. European Union: Trade in goods with United Kingdom. https://webgate.ec.europa.eu/isdb_results/factsheets/country/details_united-kingdom_en.pdf. Cited December 10, 2021.

8. Annual 2020 press highlights. https://www.census.gov/foreign-trade/statistics/highlights/AnnualPressHighlights.pdf. Cited December 2, 2021.

9. Trade in goods with United Kingdom. https://www.census.gov/foreign-trade/balance/c4120.html. Cited December 12, 2021.

10. 2021 National trade estimate report on foreign trade barriers, Office of the United States Trade Representative, Ambassador Katherine Tai. https://ustr.gov/sites/default/files/files/reports/2021/2021NTE.pdf. Cited November 10, 2021.

11. Top trading partners—October 2021. https://www.census.gov/foreign-trade/statistics/highlights/top/top2110yr.html. Cited December 20, 2021.
